# Evidence for independent representational contents in inhibitory control subprocesses associated with frontoparietal cortices

**DOI:** 10.1002/hbm.26135

**Published:** 2022-10-31

**Authors:** Negin Gholamipourbarogh, Filippo Ghin, Moritz Mückschel, Christian Frings, Ann‐Kathrin Stock, Christian Beste

**Affiliations:** ^1^ Cognitive Neurophysiology, Department of Child and Adolescent Psychiatry, Faculty of Medicine TU Dresden Dresden Germany; ^2^ University Neuropsychology Center, Faculty of Medicine TU Dresden Dresden Germany; ^3^ Cognitie Psychology University of Trier Trier Germany

**Keywords:** EEG signal decomposition, ICA, inhibitory control, MVPA, source localization

## Abstract

Inhibitory control processes have intensively been studied in cognitive science for the past decades. Even though the neural dynamics underlying these processes are increasingly better understood, a critical open question is how the representational dynamics of the inhibitory control processes are modulated when engaging in response inhibition in a relatively automatic or a controlled mode. Against the background of an overarching theory of perception‐action integration, we combine temporal and spatial EEG signal decomposition methods with multivariate pattern analysis and source localization to obtain fine‐grained insights into the neural dynamics of the representational content of response inhibition. For this purpose, we used a sample of *N* = 40 healthy adult participants. The behavioural data suggest that response inhibition was better in a more controlled than a more automated response execution mode. Regarding neural dynamics, effects of response inhibition modes relied on a concomitant coding of stimulus‐related information and rules of how stimulus information is related to the appropriate motor programme. Crucially, these fractions of information, which are encoded at the same time in the neurophysiological signal, are based on two independent spatial neurophysiological activity patterns, also showing differences in the temporal stability of the representational content. Source localizations revealed that the precuneus and inferior parietal cortex regions are more relevant than prefrontal areas for the representation of stimulus–response selection codes. We provide a blueprint how a concatenation of EEG signal analysis methods, capturing distinct aspects of neural dynamics, can be connected to cognitive science theory on the importance of representations in action control.

## Graphical abstract

We provide evidence for independent representational contents in inhibitory control subprocesses associated with frontoparietal cortices. The results provide insights into a new level of complexity underlying inhibitory control processes. We provide a blueprint how a concatenation of EEG signal analysis methods, capturing distinct aspects of neural dynamics, can be connected to cognitive science theory on the importance of representations in action control.

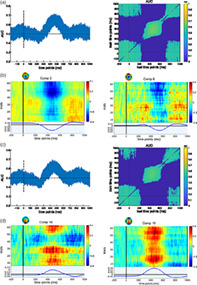

## 1 INTRODUCTION

Inhibitory control processes have intensively been studied in cognitive science, and there are many different facets of inhibitory control (Bari & Robbins, 2013). One of the most important modulating factors of inhibitory control relates to the degree of automaticity with which a response is executed. Whenever there is a strong tendency to execute a response (on a “Go” stimulus), response inhibition (on a “Nogo” stimulus) becomes more demanding and error‐prone (Dippel et al., 2016; Dippel et al., 2017; Helton, 2009; Young et al., 2018). There is a direct interrelation between the degree of automaticity to execute responses and the success of inhibitory control processes (Vahid et al., 2022; Young et al., 2018). However, cognitive science research in the field of response inhibition has long been lacking stringent experimental control over the degree of automaticity and its effects on response inhibition processes with the consequence that the underlying neural dynamics is still not understood.

From a cognitive science point of view, automaticity refers to processes that require few cognitive resources to be carried out and are difficult to terminate (Sherman et al., 2008; Ulrich et al., 2015). Opposed to this, controlled processes are voluntarily initiated processes that require a deliberate allocation of cognitive resources (Sherman et al., 2008; Ulrich et al., 2015). To experimentally manipulate the relative contribution of automated or controlled response tendencies are evident, two well‐known experimental psychology paradigms to examine action control can be combined—the Simon task (Keye et al., 2013) and the Go/Nogo task. In a Simon task, people perform a choice task in which stimuli are presented carrying task‐relevant and irrelevant features. The task‐relevant feature determines the response; the task‐irrelevant stimulus feature is not response‐relevant. Nevertheless, it can facilitate the selection of the correct response by activating the same response as the relevant stimulus feature (congruent trial), but it can also diminish the ability to select the correct response by eliciting a response tendency other than the correct response (incongruent trial) (Keye et al., 2013; Vahid et al., 2020). According to the “dual route model” (De Jong et al., 1994) (for criticism, see Hommel & Wiers, 2017), the unconditionally automatic process is described as the “direct route,” while the “indirect route” describes the conditional selection of the stimulus features that indicate the appropriate response (Hommel, 2011). In incongruent Simon trials, the automatic and controlled routes are activated and induce a conflict (Chmielewski & Beste, 2017; Hommel, 2011; Keye et al., 2013), which increases the contribution of controlled response selection processes. Using a Simon‐Go/Nogo task, it has been shown that there is a lower rate of false alarms (i.e., erroneous responses in Nogo trials) when a Nogo stimulus is embedded in incongruent than congruent Simon task trials (Chmielewski et al., 2018; Chmielewski et al., 2020; Chmielewski & Beste, 2017; Opitz et al., 2019; Wendiggensen et al., 2022). Previous studies investigated the neural dynamics underlying the interplay of automatic and controlled processes during response inhibition (Chmielewski et al., 2018; Chmielewski et al., 2020; Chmielewski & Beste, 2017; Opitz et al., 2019; Wendiggensen et al., 2022). Numerous studies used event‐related potential (ERP) analysis to investigate cognitive processes correlates during the Simon task (Cespón et al., 2020). For this purpose, studies focused on different ERPs such as the fronto‐central N200, the medial frontal negativity, the LRP, and the P3 (Cespón et al., 2020). Others analysed the spectral power obtained in different experimental conditions of the Simon task. These studies mainly focused on the theta band, which was related to cognitive control. Results showed the increase of theta‐band activity over the mid‐frontal region in incompatible compared to compatible trials of the Simon task. It is also suggested that increased midfrontal theta activity represents a neural marker of cognitive control. In similar vein, ERPs (in the N2 and the P3 time window) as well as theta and alpha band activity modulations have a long research tradition in the analysis of neural processes underlying response inhibition (Huster et al., 2013). Importantly, while research in the tradition of ERP and event‐related oscillations readouts were able to outline the time and intensity of neural processes during conflict monitoring (e.g., in the Simon task) and inhibitory control (e.g., in the Go/Nogo task), these methods cannot spot light on the neural processes underlying the management of *representations* during cognitive operations. Relatedly, a critical question is still whether and how the *representation* of the inhibitory control processes is differentially modulated when engaging in response inhibition in a relatively automatic or a controlled mode.

One approach to answer that question is to use multivariate pattern analysis (MVPA) methods and apply these to EEG data. Opposed to more classical methods such as time‐frequency decomposition and ERPs, EEG‐based MVPA can decode the representational difference between experimental conditions based on the observed neural patterns (Carlson et al., 2019; Fahrenfort et al., 2018; King & Dehaene, 2014). In the current study, we use EEG‐based MVPA to examine whether the representation of the inhibitory control processes is differentially modulated when engaging in response inhibition in a relatively automatic or a controlled mode. In particular, we apply temporal generalization MVPA (Fahrenfort et al., 2018; King & Dehaene, 2014; Petruo et al., 2021; Prochnow et al., 2021; Takacs, Mückschel, et al., 2020) because this approach is suitable to examine when and for how long representations are differentially activated between conditions (King & Dehaene, 2014; Petruo et al., 2021). Critically, however, previous lines of research have shown that the effects of a relatively automatic or controlled response selection mode affect specific fractions of information being concomitantly coded in the EEG signal (Chmielewski et al., 2018). This was made possible by applying a temporal signal decomposition method—residue iteration decomposition (RIDE) (Ouyang et al., 2015). Invented to control intra‐individual variability in EEG signals (Ouyang et al., 2011), this method can also distinguish between fractions of information coded in the EEG during the inhibitory control (Mückschel et al., 2017), because RIDE decomposes the EEG signal into theoretically meaningful distinct activity clusters: The S‐cluster has been conceptualized to reflect the stimulus‐related activity. The C‐cluster has been conceptualized to reflect the mapping of stimuli to the response (Ouyang et al., 2017; Takacs, Zink, et al., 2020). The C‐cluster information is differentially modulated by a relatively automatic or a controlled mode during the response inhibition (Chmielewski et al., 2018). Therefore, we use temporalization MVPA in RIDE‐decomposed data to examine whether the representation of the inhibitory control processes is differentially modulated when engaging in response inhibition in a rather automatic or a controlled mode. Through the combination of RIDE‐decomposition and temporal generalization MVPA, it will be possible to gain insights into the temporal representational dynamics of two intermingled yet separable information processing levels during inhibitory control as modulated by a more controlled or more automated response execution mode.

However, the question is whether this combination of methods is sufficient to provide fine‐grained insights into the neural processes involved? In fact, this may not be case when considering one important cognitive theory on goal‐directed behaviour and perception‐action integration. A well‐established theoretical framework (i.e., theory of event coding [TEC]) suggests that stimulus–response mappings are implemented through so‐called event files (Hommel, 2004; Hommel et al., 2001) and several lines of research have provided evidence that event‐file processes during response selection and inhibition are reflected by neural dynamics captured in the above‐mentioned C‐cluster (Opitz et al., 2020; Prochnow et al., 2021; Takacs, Zink, et al., 2020). According to TEC, event file processing requires spatially distributed activity (Hommel, 2004) to accomplish the integration of perceptual and motor processes (also known as stimulus–response mapping). Crucially, the spatial properties of neural activity are not considered in the RIDE algorithm (Ouyang et al., 2015). Since the C‐cluster very likely reflects event file dynamics (Opitz et al., 2020; Prochnow et al., 2021; Takacs, Zink, et al., 2020) it is possible that spatially distinct neural activity profiles can further be dissociated within RIDE cluster activity. To examine this question, it is conceptually necessary to spatially decompose activity captured by the different RIDE clusters (i.e., the S‐cluster and especially so the C‐cluster). One means to do so is to apply independent component analysis (ICA) on RIDE‐decomposed data. Using ICA, it is possible to obtain independent activity profiles with their spatial distribution (i.e., components, ICs) of neural activity (Delorme et al., 2012; Huster et al., 2015; Huster & Raud, 2018). Applying ICA, we explore whether there are spatially independent activity profiles constituting neural dynamics captured using RIDE. According to TEC, this should be case for stimulus response mapping processes that are likely to be captured by the RIDE C‐cluster. For each of the isolated spatial activity profiles, we then perform temporal generalization MVPA to examine whether the isolated ICs also show distinct temporal profiles of the representational content. If this is the case, this will give rise to the assumption that distinct functional neuroanatomical structures are involved in the differential concomitant representation of stimulus–response mapping codes and its temporal profile in response inhibition in a relatively automatic or a controlled mode. To objectify this, we apply source localization to those time periods in which significant decoding of the representational content in an independent component (IC) is possible to delineate which functional neuroanatomical structures are associated with the processes (Petruo et al., 2021). In summary, the current study combines complementary neurophysiological signal decomposition methods (i.e., RIDE and ICA) with representation decoding methods (i.e., temporal generalization MVPA) and source localization to obtain fine‐grained insights into neural processes underlying an essential modulator of response inhibition processes.

## 2 MATERIALS AND METHODS

### 2.1 Participants

A sample of *N* = 40 healthy adult participants (23.9 ± 2.5 years of age; 23 females) was randomly drawn from a larger, existing database. All participants were free of any medication, showed normal or corrected‐to‐normal vision, and had no history of neurological psychiatric or disorders. We performed neuropsychological assessments including the Beck Depression Inventory (3.46 ± 2.85) and the Mini Mental Status Examination (29.26 ± 0.86). The sample size used is comparable to previous studies using the same task (Chmielewski et al., 2018; Chmielewski et al., 2020; Chmielewski & Beste, 2017; Opitz et al., 2019; Wendiggensen et al., 2022) or studies using MVPA in combination with RIDE (Prochnow et al., 2021; Takacs, Mückschel, et al., 2020). All participants provided written informed consent. The study was approved by the ethics committee of the TU Dresden.

### 2.2 Task

**FIGURE 1:**
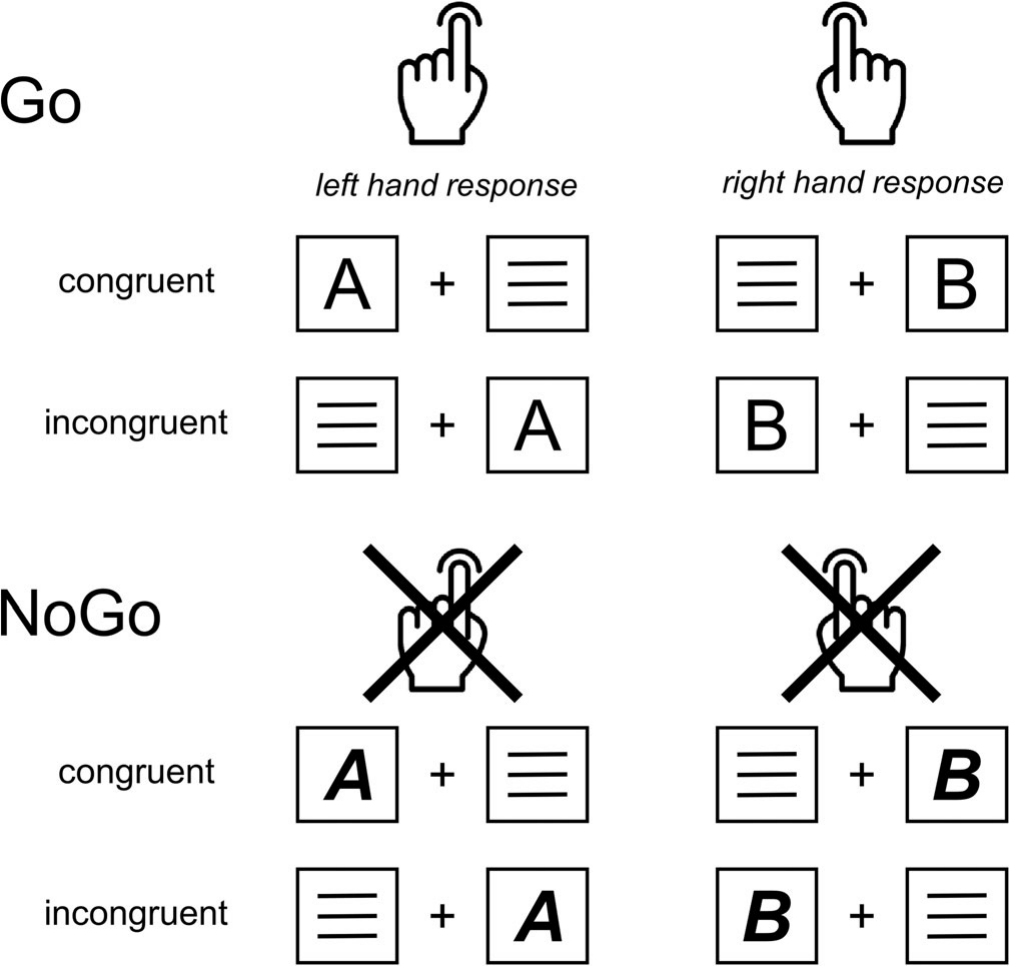
Simon‐NoGo paradigm. Shown are all possible stimulus–response combinations for the Go condition and the NoGo condition. For the behavioural and EEG data analysis, left‐ and right‐hand responses were not distinguished

As outlined in the introduction, we use a combined Simon‐Go/NoGo task to experimentally vary the impact of more automated or controlled response selection tendencies on the response inhibition (Chmielewski et al., 2018; Chmielewski et al., 2020; Chmielewski & Beste, 2017; Opitz et al., 2019; Wendiggensen et al., 2022). The structure of the task is shown in Figure 1.

White stimuli were presented on a black background (57 cm viewing distance). A fixation cross was presented continuously in the centre of the screen, and a white box was presented at the same vertical height to the left and right of the fixation cross (1.1° viewing distance). Each trial began with the presentation of a letter (i.e., “A” or “B”) for 200 ms in one of the white boxes. Subjects had to respond within 1700 ms (for Go trials). An incorrect response within this time window was coded as an error, and if no response was made, the trials were coded as a miss. A letter stimulus was either presented in normal font or in bold font on each trial. Participants were asked to respond as quickly as possible to letters in a standard font (Go trials), while they had to inhibit their responses when the letter stimuli were presented in combined bold and italic font (NoGo trials). A left‐hand response was required when an “A” was displayed in the Go trials, while a right‐hand response was required when a “B” was displayed. These responses were required regardless of the spatial position of the letter stimuli in the left or right white frame on the screen. This created a congruent Go condition in which the stimuli were presented on the side of the hand making the response, and a Go incongruent condition in which the stimuli were presented on the side opposite to the hand executing the response. In the NoGo trials (bold and italic letters), the left side “A” and the right side “B” represented congruent NoGo trials, while the left side “B” and the right side “A” represented incongruent NoGo trials. For NoGo trials, any response before 1700 ms resulted in the trial being coded as a false alarm. Each trial ended after 1700 ms if no response was registered beforehand. The inter‐trial interval was jittered between 1300 and 1700 ms. The experiment consisted of 720 trials (70% [504 trials] Go and 30% [216 trials] NoGo trials), of which 50% were congruent, and 50% were incongruent. The experiment was divided into 6 blocks of 120 trials each. The trial types (congruent and incongruent Gos or congruent and incongruent NoGos) were presented randomly, ensuring that all conditions were evenly distributed across the blocks. Before the experiment, each subject was trained on the task using 40 trials.

### 2.3 EEG recording and pre‐processing

**FIGURE 2:**
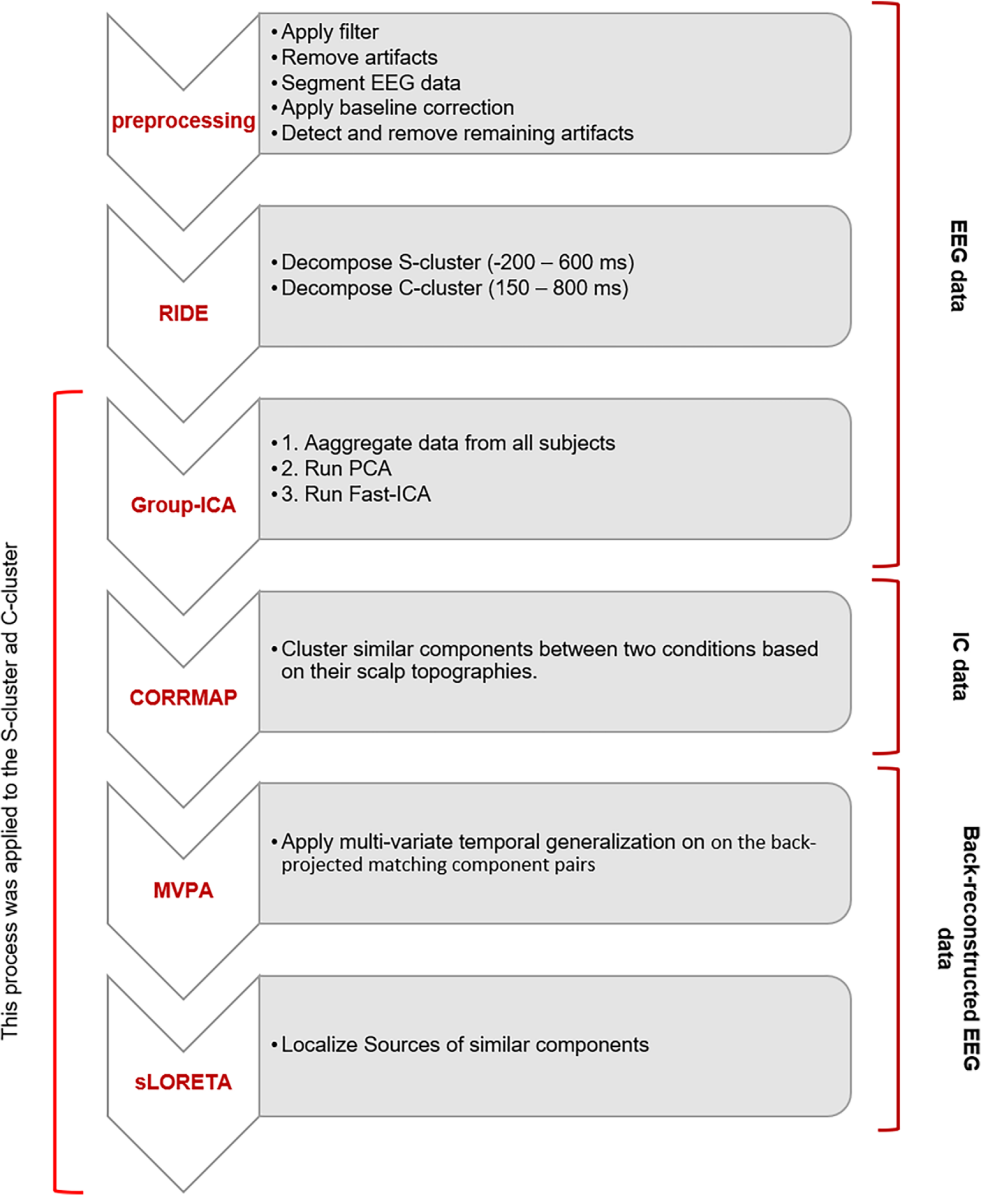
Flowchart delineating the concatenation of the used EEG signal analysis methods

The EEG signal was recorded with a QuickAmp amplifier (Brain Products GmbH, Gilching, Germany) using 60 Ag/AgCl electrodes, with the reference electrode positioned at Fpz (*θ* = 90, *φ* = 90) and the ground electrode positioned at *θ* = 58, *φ* = 78. The impedance of the electrodes was kept below 10 kΩ. The Brain Vision Analyzer software pre‐processed all data sets (Version 2.2; Brain Products GmbH). The data were downsampled to 256 Hz (recording frequency was 500 Hz), and band‐pass filtered (IIR) was applied between 0.5 and 40 Hz. When necessary, noisy channels were eliminated, and recordings were re‐referenced to the total activity of all electrodes. By manually evaluating each raw data set, technical artefacts were removed. Technical artefacts refer to noise associated with a device's electrical system or environment. Artefacts that occur periodically, such as cardiovascular artefacts, eye blinks, and saccades, were detected visually and corrected using an ICA (Infomax algorithm). After that, the exported data were segmented into epochs from −2000 to 2000 ms and locked to the stimulus onset in the different trial conditions (congruent or incongruent Go and NoGo trials). The Go and NoGo conditions segmentation was carried out for congruent and incongruent trials separately. Then, an automated artefact rejection was carried out discarding all EEG epochs showing (with amplitudes greater than 100 V and less than −100 V, and activity less than 0.5 V over a 100 ms time interval). After this step, a current source density transformation was applied to allow a reference‐free evaluation of the EEG data. Finally, a baseline correction was applied from −300 to 0 ms. After data pre‐processing, one participant was removed because the number of final trials was insufficient for further analysis. In the final step, the data were divided into two different groups of trials and compared between congruent and incongruent conditions: (i) Go trials and (ii) Nogo trials. All procedures are shown in Figure 2. The data analysis was conducted on each group separately with a focus on the comparison (ii), because the goal of the current study is to examine effects of the automated or controlled response execution mode on response inhibition. The results of the other comparison are shown in the supplemental material.

### 2.4 Temporal signal decomposition (RIDE)

As shown in Figure 2, the RIDE approach (Ouyang et al., 2015) was used to decompose the segmented single‐trial EEG data (RIDE toolbox available at http://cns.hkbu.edu.hk/RIDE.htm). The RIDE method can detect separate component clusters from ERP data associated with different cognitive processes based on their temporal variability. A component cluster is a collection of EEG components locked to the same time event. Based on their timing and timing variability, RIDE is used to decompose single‐trial level data into components with fixed and variable latency. Decomposed clusters represent a stimulus‐locked component cluster (S‐cluster), which is expected to include brain processes, which reveal latencies locked to the stimulus onset and are mostly unrelated to response speed. Then, there is a response‐locked component cluster (R‐cluster) intended to include motor‐related processes like motor preparation and execution, and an intermediate, nonmarker‐locked component cluster (C‐cluster) which is mostly attributed to latency‐variable processes and is considered to be connected to central cognitive processes, like stimulus evaluation and response selection (Ouyang et al., 2011). RIDE separates clusters using temporal markers, or “latencies” (“SL” and “RL”), that are derived according to the stimulus and response onsets, respectively. The C‐cluster (“CL”) time markers change with time and must be estimated and improved iteratively. RIDE subtracts C and R from each trial to estimate S. The residuals from all trials are then aligned to the latency SL, yielding S as the median waveform for all time points. The C‐cluster and the R‐cluster are calculated in the same way. Thus, the general procedure of RIDE for separating S, C, and R component clusters involves the following steps: (1) initial estimation of the latency of C in single trials; (2) decomposing S, C, and R, based on stimulus onsets, RTs, and latencies of C in single trials; (3) using C, separated in (2) as a template to re‐estimate the latencies of C by template matching; (4) Iteration of Steps (2) and (3) until convergence (Ouyang et al., 2015). However, in the current study, no R‐cluster was estimated in the RIDE decomposition because responses occur with too low frequency to allow a reliable estimation of the R cluster. Also, in the Nogo condition, a response is not available as a marker (Ouyang et al., 2013). Thus, only the S‐cluster and the C‐cluster are computed in Go/Nogo task, and the C‐cluster reflects motor‐response related processes. The RIDE algorithm considers each electrode channel separately and does not consider the scalp activity profile (Ouyang et al., 2015). A time window function is used to extract the waveform of each RIDE component. This time window must be set manually. The S‐time cluster's range of interest was −200 to 600 ms around stimulus initiation, whereas the C‐time cluster's range was 150–800 ms after stimulus onset. The time windows chosen correspond and large overlap with time windows that have been used for RIDE decomposition using the same task in previous publications (Chmielewski et al., 2018; Chmielewski et al., 2019; Wolff et al., 2019) to ensure comparability of the procedures taken.

### 2.5 Spatial signal decomposition (ICA)

As shown in Figure 2, using Group‐ICA, RIDE‐cluster data were decomposed into sub‐components. ICA does not naturally generalize to a method suitable for drawing inferences about groups of subjects because it estimates different sets of components in different orders and scales for each participant or run, so it is not immediately clear how to draw inferences about group data using ICA. So, an approach would be creating aggregate data from all subjects and estimating group ICs. The Group‐ICA method extracts ICs of the group data (Calhoun et al., 2009) using the equation C = WX that attempts to estimate independent brain components (C), from homogeneous neurophysiological activity of RIDE decomposed data (X) across subjects. W represents the de‐mixing matrix in this equation (Hyvärinen & Oja, 2000). Group‐ICA contains four steps:

The first step is employing a data reduction on pre‐processed data using principal component (PC) analysis to reduce computational effort in the following ICA decomposition steps. In the current study, we considered a threshold for the percentage of eigenvalues retaining (98%), and the results showed that by selecting 20 PCs, the amount of this retaining for all subjects was higher than the threshold. After that, data from all subjects' observations are aggregated. The aggregate set is created by concatenating individual PCs. Then, ICA (Fast‐ICA method (Hyvärinen & Oja, 1997)) calculates the ICs of the group data. In the final step, we perform a back‐reconstruction to retrieve individual ICs. The EEGIFT toolbox (http://icatb.sourceforge.net/EEGIFT) was used to execute group‐ICA (Eichele et al., 2011; Rachakonda et al., 2011). It was applied to the C‐cluster and S‐cluster for congruent and incongruent conditions separately. Estimated group ICs for congruent and incongruent conditions are generated with different order and scales. So, they need to be matched across these conditions to allow comparison between two different conditions. For this purpose, an approach would be combining group ICs across conditions with clustering techniques. CORRMAP (http://www.debener.de/corrmap) (Viola et al., 2009) is a semi‐automated clustering approach that clusters components solely based on the correlation of their inverse weights using an IC template specified by the user. We used 40 different clusterings, with each of the group components serving as the initial core for each cluster (i.e., components of congruent and incongruent conditions). The homogeneous ICs from two congruent and incongruent conditions whose weighting matrices had a correlation coefficient greater than a set threshold were then identified. The threshold of the correlation between IC topographies was set at 0.85. To find as many similar components as possible for each template, the maximum number of represented components of each group in each cluster was set to three. It prevented CORRMAP clustering from finding similar overlapping components.

### 2.6 Multivariate pattern analysis

Using the MVPA light toolbox (refer to Figure 2), we performed MVPA on the back‐projected matching component pairs (CORRMAP results). Each pair was investigated using two analyses at the single‐subject level: a binary classification (for congruent and incongruent trials) across time to identify time points with different patterns between IC pairs and a temporal generalization analysis to characterize the temporal dynamics of the representational content at the component ERP level. The amplitudes of EEG channels at individual electrodes were utilized as classification features, creating 60 features in both conditions. To avoid an overfitting problem, under‐sampling was employed to balance the number of trials in two classes before the MVPA for each individual and IC pair. An SVM classifier was trained and verified for each individual and each IC pair separately for classification and temporal generalization analysis (for the S‐cluster and C‐cluster ICs). A fivefold cross‐validation was applied twice for both analyses, which means that the classifier was trained on 80% of the data and tested 20% of the data, and the procedure was repeated until all data chunks were tested. For each trial, only signals from 0 to 1000 ms after stimulus onset were used for MVPA. The area under the ROC curve (AUC) was employed to evaluate classification accuracy. The average of test folds was used to get the final performance metric. Wilcoxon tests against chance level (AUC = 0.5) were performed for each time sample across subjects to identify the time points showing significant classification performance. Cluster‐based permutation was used as a correction for multiple comparisons. The sum of Wilcoxon test values within time points was used to calculate cluster‐level statistics. This process was carried out 1000 times.

### 2.7 Source localization analysis

Source localization analyses were performed using the software package for standardized low‐resolution tomography (sLORETA) (Pascual‐Marqui, 2002). The source localization was conducted for the ICs constituting the RIDE S‐cluster and C‐cluster activity, and in which temporal stability (i.e., off‐diagonal activity) of representational content was revealed using MVPA. To this end, we compared congruent and incongruent trials (i.e., congruent minus incongruent) in these RIDE‐cluster‐ICs using sLORETA's built‐in randomization tests based on statistical nonparametric mapping (SnPM) to correct for multiple comparisons (*p* < .05). For this, 2000 permutations were used. sLORETA is based on a realistic head model (Fuchs et al., 2002) and estimates sources without localization error (Sekihara et al., 2005). For this purpose, the intracerebral volume is divided into 6239 voxels with 5 mm spatial resolution. Based on the MNI152 template, the standardized current density at each voxel is calculated to localize the sources. Using brain stimulation‐EEG and fMRI/EEG or structural imaging, sLORETA yields reliable source estimations (Sekihara et al., 2005). Since the focus of the current work in on response inhibition and the effects of more automated (congruent trials) or controlled (incongruent trials) response selection modes, we only performed the sLORETA analysis for Nogo trials.

## 3 RESULTS

### 3.1 Behavioural data

The *t* test for correct‐Go RTs indicated a main effect of congruency (*t*(38) = −3.84; *p* < .001; *d* = .62), with faster RTs in congruent (539.18 ± 12.40 ms) trials than in incongruent trials (549.63 ± 11.24 ms). For the Go condition, a dependent *t* test for accuracy revealed there were no significant difference between congruent (97.77 ± .25) and incongruent (97.51 ± 0.25) conditions (*t*(38) = 1.107; *p* = .27; *d* = .18).

In the NoGo condition, however, there was a significant difference between congruent and incongruent trials (*t*(38) = −2.86; *p* = .07; *d* = −.46), with incongruent trials (95.32% ± 0.76) having greater accuracies (significantly more correct omissions) than congruent trials (93.9% ± 0.92). Because the accuracy variables failed to meet the normality assumption (*p* < .05), post hoc nonparametric tests were used to confirm the results. The difference between the NoGo congruent and NoGo incongruent conditions was statistically significant according to the Wilcoxon signed‐rank test (*z* = −2.7; *p* = .007).

### 3.2 Neurophysiological data (Nogo trials)

The Group‐ICA analysis was used to estimate and aggregate back‐reconstructed sub‐components of congruent and incongruent Nogo trials in the −200 to 1000 ms time interval. This was done for the RIDE C‐ and S‐cluster components separately. Supplementary Figure S1 shows the scalp topography plots of the group ICs for congruent and incongruent Nogo trials in the S‐cluster and the C‐cluster.

**FIGURE 3:**
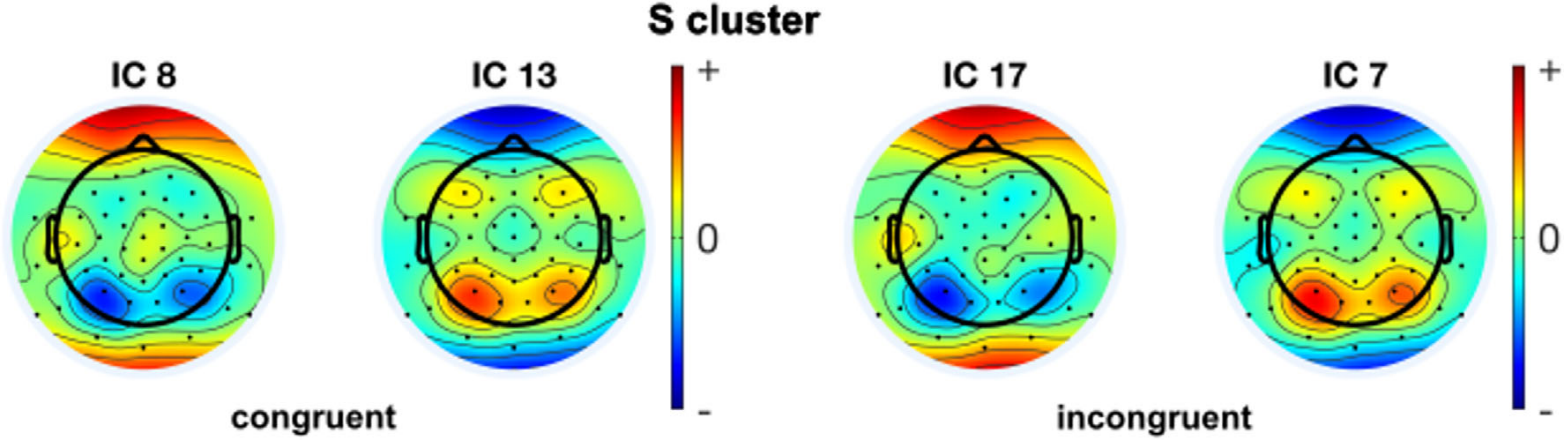
Results from the CORRMAP processing step for the RIDE S‐cluster in the congruent (left) and incongruent (right) conditions. The scalp topographies reveal the weighting matrices of each IC

**FIGURE 4:**
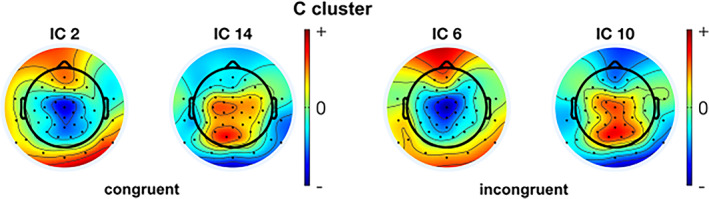
Results from the CORRMAP processing step for the RIDE C‐cluster in the congruent (left) and incongruent (right) conditions. The scalp topographies reveal the weighting matrices of each IC

The IC scalp maps of congruent and incongruent conditions for the Nogo trials were investigated separately using CORRMAP. This clustering was applied to RIDE S‐clusters and C‐cluster IC‐components independently. It aggregated each component with the same weight distribution on the channel space as its corresponding condition components (from both conditions) into a single cluster using the correlation threshold of 85%. For the Nogo trials, the analysis of the final clusters revealed two pairs of homogeneous ICs between congruent and incongruent conditions for the S‐cluster and two pairs of homogeneous ICs between congruent and incongruent conditions for the C‐cluster. The corresponding ICs are shown in Figures 3 and 4, respectively. The numbers of the homogenous ICs and the similarity represented by the correlation of ICA inverse weights of each pair for S and C clusters between congruent and incongruent trials are presented in Tables 1 and 2, respectively.

**TABLE 1:** Similar components for the S‐cluster in the congruent and incongruent NoGo conditions according to the CORRMAP analysis. The correlation coefficient shows the similarity between scalp topographies of each IC pair. The scalp topography plots of the original ICs for the congruent and incongruent NoGo conditions are shown in the supplemental material

New component number	Original IC number for congruent trials	Original IC number for incongruent trials	Correlation between scalp topographies
1	8	17	0.90
2	13	7	0.97

**TABLE 2:** Similar components for the C‐cluster in the congruent and incongruent NoGo conditions according to the CORRMAP analysis. The correlation coefficient shows the similarity between scalp topographies of each IC pair. The scalp topography plots of the original ICs for the congruent and incongruent NoGo conditions are shown in the supplemental material

New component number	Original IC number for congruent trials	Original IC number for incongruent trials	Correlation between scalp topographies
1	2	6	0.91
2	14	10	0.91

**FIGURE 5:**
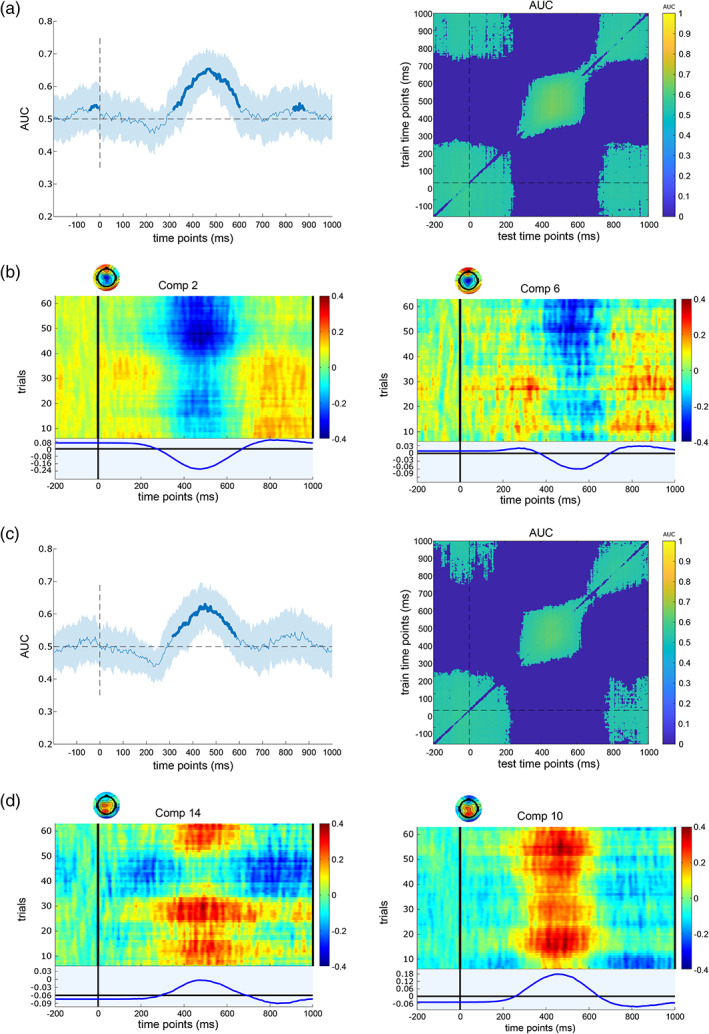
MVPA results for selected IC pairs of the RIDE S‐cluster. (a) Shows the binary classification performance (left) and the temporal generalization (right) of the first independent component. For the binary classification, the shaded error bars represent standard deviation. (b) Illustrates the event‐related potential (ERP) image (scalp topography, trial activity, and ERP signal) of the first selected IC for two congruent (left) and incongruent (right) NoGo conditions in the time between −200 and 1000 ms. Colour bars show trial activities. Plot (c) shows the binary classification performance (left) and the temporal generalization (right) of the second independent component. The shaded error bars represent standard deviation for the binary classification. (d) Shows the ERP image (scalp topography, trial activity, and ERP signal) of the second IC for two congruent (left) and incongruent (right) conditions in the −200 to 1000 ms time interval. Colour bars show trial activities

**FIGURE 6:**
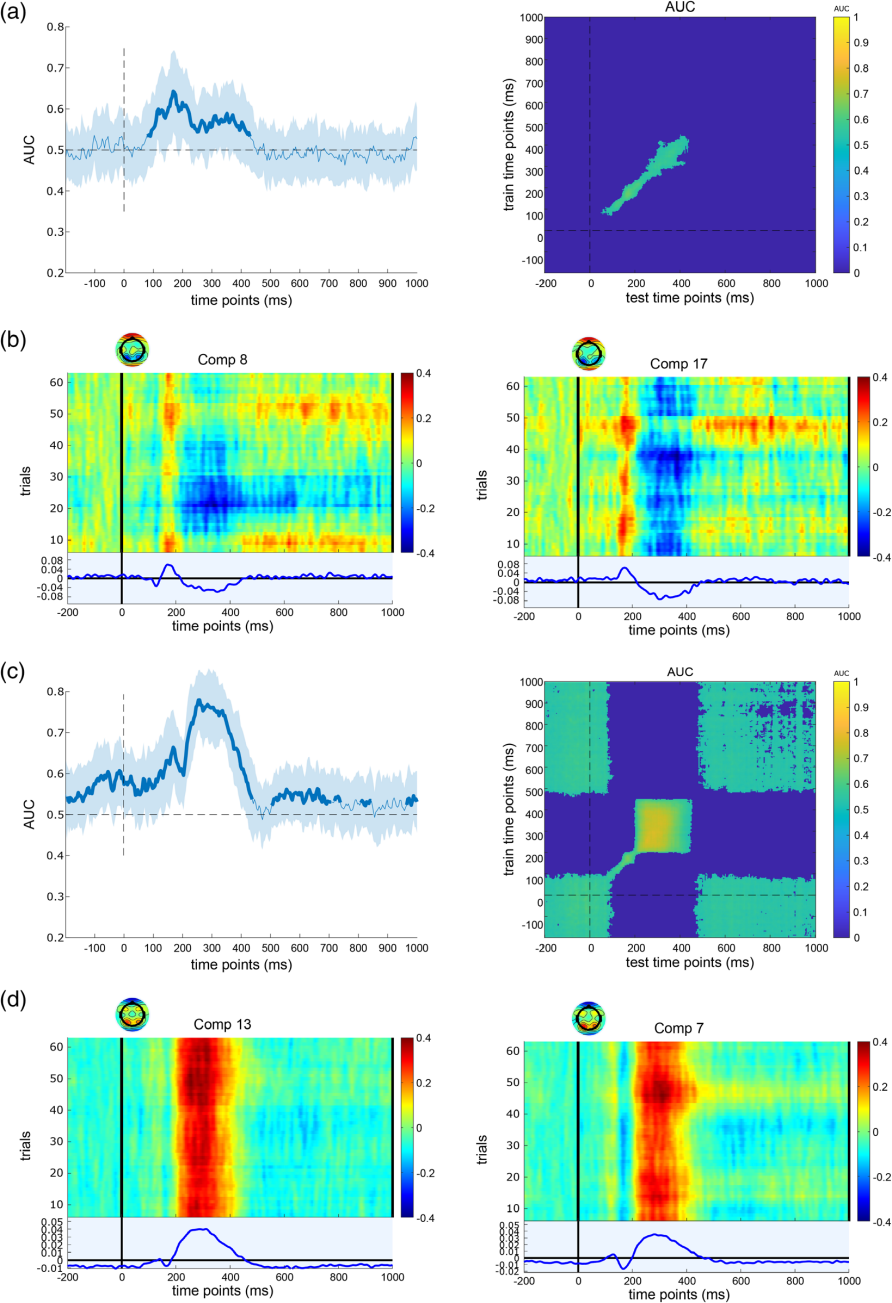
MVPA results for selected IC pairs of the RIDE C‐cluster. (a) Shows the binary classification performance (left) and the temporal generalization (right) of the first independent component. For the binary classification, the shaded error bars represent standard deviation. (b) Illustrates the event‐related potential (ERP) image (scalp topography, trial activity, and ERP signal) of the first selected IC for two congruent (left) and incongruent (right) NoGo conditions in the time between −200 and 1000 ms. Colour bars show trial activities. Plot (c) shows the binary classification performance (left) and the temporal generalization (right) of the second independent component. The shaded error bars represent standard deviation for the binary classification. (d) Shows the ERP image (scalp topography, trial activity, and ERP signal) of the second IC for two congruent (left) and incongruent (right) conditions in the −200 to 1000 ms time interval. Colour bars reveal trial activities

After that, the back‐projected ERP levels were also investigated to evaluate the signal‐to‐noise ratio of extracted components and compare them between congruent and incongruent conditions. For the S‐cluster and C‐cluster, the MVPA was applied to each extracted homogeneous IC pair independently. For the MVPA a contrast (comparison) was performed between congruent and incongruent Nogo trials. Figures 5 and 6 reveal the performance of binary classification between congruent and incongruent Nogo trials over time and the temporal generalization matrix for the S‐cluster and the C‐cluster, respectively.

Regarding the S‐cluster results (Figure 5), the classification AUC results of IC pair 1 were significant (with a peak of 64.36%) in the time interval between 100 and 400 ms after stimulus onset. The temporal generalization cluster was observed in this time interval. However, the ERP data for both congruent and incongruent Nogo conditions showed a high signal‐to‐noise ratio. For the IC pair 2, the AUC curve of the binary classification was significant between 200 and 450 ms (a peak of 77.9%) after stimulus presentation and an off‐diagonal activity (temporal generalization) of approximately 200 ms in this time interval. The activity of back‐projected EEG also had a positive amplitude between 200 and 400 ms.

For the C‐cluster (Figure 6), the binary classification performances of IC pair 1 were significantly higher (with the peak of 65.36%) than the chance level (AUC > .5, *p* < .05) in the time interval between 300 and 600 ms post‐stimulus. Temporal generalization clusters almost centralize around 500 ms post‐stimulus onset. Further analysis of ERP data for both congruent and incongruent conditions revealed negative activities in the 400–600 ms time range, which is entirely within the predetermined C‐cluster time range. The MVPA results for the IC pair 2 also showed significant differences between conditions for the classification (with the peak of 63.04%) and the temporal generalization cluster also was activated in the 300–600 ms time interval. The activity of the ERP data was positive for this IC pair.

**FIGURE 7:**
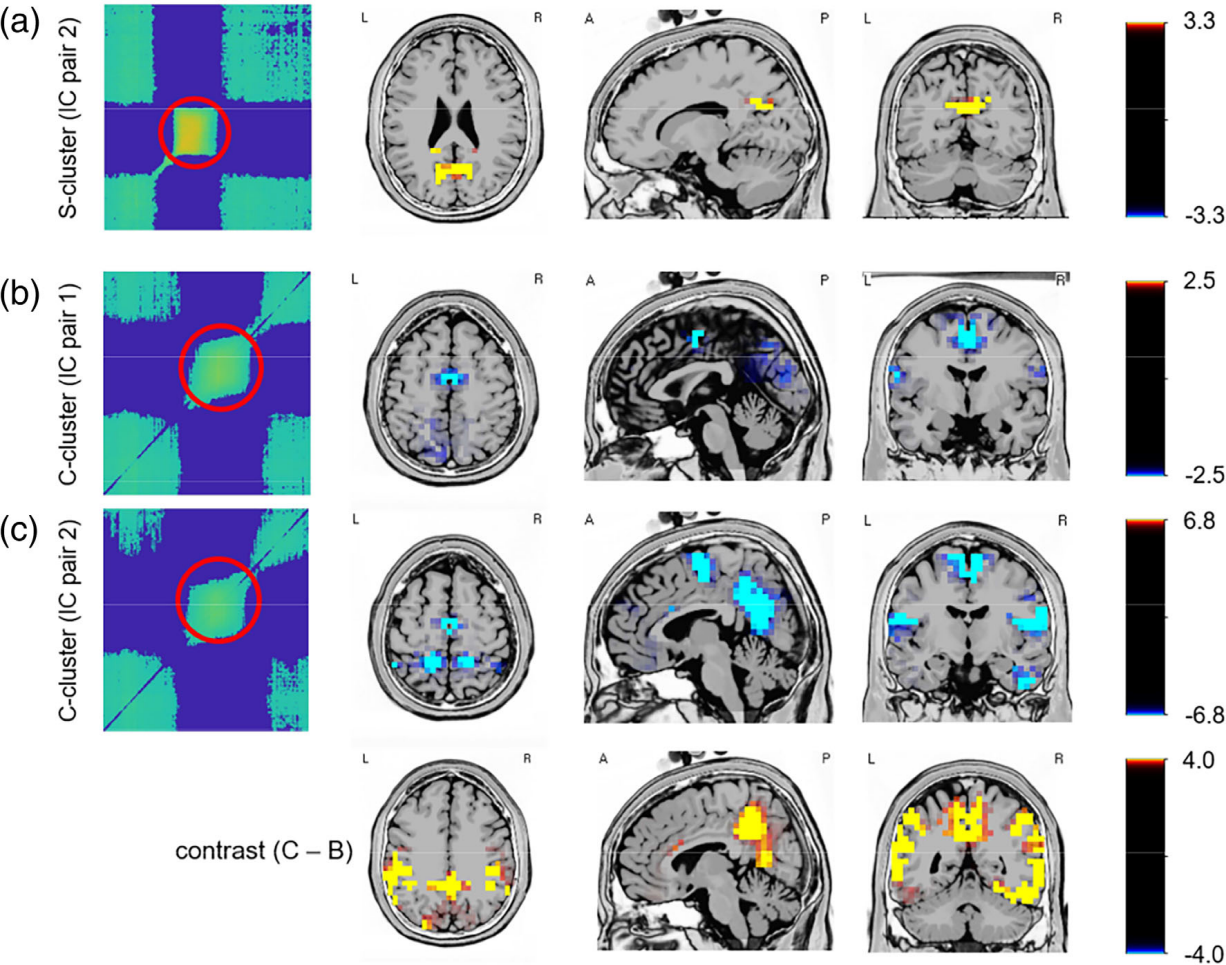
Results from the source localization using sLORETA. (a) Shows the source underlying activity captured by the S‐cluster IC pair in two in the time interval revealed temporal generalization (stability) of the representational content. (b) Shows the source underlying activity captured by the C‐cluster IC pair in one in the time interval revealed temporal generalization (stability) of the representational content. (c) Shows the source underlying activity captured by the C‐cluster IC pair in two in the time interval revealed temporal generalization (stability) of the representational content. Also, the contrast between B and C is shown. Only significant brain regions are shown the sLORETA colour bar denotes the critical *t*‐values after randomization tests

The results of the source localization analysis of the Nogo trials datasets are shown in Figure 7.

For the S‐cluster and IC pair 1, temporal generalization was hardly evident, which is why the sLORETA analysis was not conducted. For the IC pair 2, temporal generalization was evident between 200 and 400 ms after stimulus presentation. For this time interval, the sLORETA analysis contrasting congruent and incongruent Nogo trials (contrast: congruent Nogo minus incongruent Nogo) revealed activity modulations in the posterior cingulate gyrus (the caudal part of the cingulate cortex, located posterior to the anterior cingulate cortex [ACC]) and the precuneus, the medial parietal cortex (Brodmann area [BA] 31 and 7, respectively) (Figure 7, top row).

For the C‐cluster data, and IC pair 1 (Figure 7, middle row) showing temporal generalization between 300 and 600 ms post‐stimulus, the sLORETA analysis (contrast: congruent Nogo minus incongruent Nogo) revealed activity modulations in the ACC (BA24) and the medial frontal gyrus (BA6). For the IC pair 2 (Figure 7, bottom row), showing temporal generalization in the same time window, the sLORETA analysis (contrast: congruent Nogo minus incongruent Nogo) also revealed activity modulations in the ACC (BA24) and the medial frontal gyrus (BA6). However, strong modulations were also found in the precuneus (BA7, BA31) and bilaterally in the inferior parietal cortex (BA40). When contrasting the C‐cluster IC pair 1 and IC pair 2 in the time window between 300 and 600 ms (i.e., (IC1_congruent Nogo_ − IC1_incongruent Nogo_) − (IC2_congruent Nogo_ − IC2_incongruent Nogo_)), it is shown that activity differences were mainly due to areas in the precuneus (BA7) and the bilateral inferior parietal cortex (BA40) extending to the temporal cortices (bilaterally).

### 3.3 Neurophysiological data (Go trials)

In addition to the Nogo trial analysis, the identical IC analysis on RIDE‐decomposed data was performed for Go trials (to compare congruent and incongruent conditions). The CORRMAP analysis on data from Go trials extracted one pair of homogeneous ICs between congruent and incongruent conditions of the S‐cluster and five pairs of homogenous ICs between aforementioned conditions for the C cluster. Homogenous IC numbers and scalp topographies are shown in the supplemental Figures S5 and S6. For the S‐cluster and C‐cluster, the MVPA was applied to each extracted homogeneous IC pair of congruent and incongruent conditions separately. Figures S5 and S6 of the supplemental material present the binary classification performance over time and the temporal generalization matrix for the S‐cluster and the C‐cluster of the Go trials data group, respectively.

Regarding the S‐cluster (supplemental Figure S5), the AUC curve of the binary classification was significant for the IC pair between 100 and 400 ms (a peak of 70%) after stimulus presentation. Temporal generalization was observed in the 200–400 ms time interval. For the first two component pairs of the C‐cluster, the MVPA revealed no significant decoding results. The classification AUC results of IC pair 3 (supplemental Figure S6) revealed significant decoding (with a peak of 58%) in the time interval between 550 and 750 ms after stimulus onset and an off‐diagonal activity (temporal generalization) in the 500–800 ms time window. Also the MVPA results for the IC pair 4 showed significant differences between two congruent and incongruent conditions for the classification (with the peak around 55%). The temporal generalization cluster also was activated in the time between 350 and 600 ms. Binary classification performances of IC pair 5 were significantly higher (with the peak of 61.5%) than the chance level (AUC > .5, *p* < .05) in the time interval between 300 and 500 ms and also 650 and 800 ms post‐stimulus. Temporal generalization clusters almost centralized around 400 ms post‐stimulus representation.

## 4 DISCUSSION

The goal of the current study was to provide fine‐grained insights into the dynamics of the representational content of response inhibition processes, emphasizing how a relatively automatic versus controlled response selection mode modulates this representational content. To this end, we integrated temporal and spatial EEG signal decomposition methods before examining the representational content of the neurophysiological signals using temporal generalization MVPA. The functional neuroanatomical basis of this dynamic was examined using source localization results (sLORETA).

The behavioural data replicate previous findings using the applied paradigm (Chmielewski et al., 2018; Chmielewski & Beste, 2017). Response inhibition was better in incongruent trials than congruent trials, as indicated by the rate of false alarms. The rate of false alarms was higher in congruent than incongruent trials. This can well be explained along the lines of the dual‐route model of the Simon task. Response selection in congruent Simon task trials has been suggested to only involve the direct route, whereas response selection in incongruent trials is suggested to operate via the direct and the indirect route (De Jong et al., 1994; Keye et al., 2013). The direct route operates via automatic processes, whereas the indirect route operates via more controlled processes. Activating the automatic and controlled routes in incongruent trials induces a conflict (Chmielewski & Beste, 2017; De Jong et al., 1994; Hommel, 2011; Keye et al., 2013), which increases controlled processes. Therefore, automated response tendencies become diminished in incongruent compared to congruent trials. Reducing an automated response tendency and increasing cognitive control resources then facilitates the ability to inhibit a response (Chmielewski et al., 2018; Chmielewski et al., 2020; Chmielewski & Beste, 2017; Opitz et al., 2019; Wendiggensen et al., 2022).

Regarding the neurophysiological data, it is shown that the neural dynamics reflected by the S‐cluster and the C‐cluster data, as derived using temporal signal decomposition using RIDE (Ouyang et al., 2015), are constituted by two spatially ICs of activity. Modulations of response inhibition processes by more automated or controlled response selection modes (i.e., congruent and incongruent trials) thus rely on a concomitant coding of stimulus‐related information (S‐cluster) and rules of how stimulus information has to be related to the appropriate motor programme (C‐cluster) (Chmielewski et al., 2018). Crucially, these fractions of information are based on two distinct (independent) spatial neurophysiological activity patterns, which suggest that the coding of each of these fractions of information is accomplished in spatially distinct neural assemblies. This suggests that inhibitory control processes and their modulation by a relatively automatic or a controlled response execution context are represented in spatially distributed neural ensembles (Christophel et al., 2017; Land et al., 2013). Considering the TEC‐framework (Hommel, 2004), this pattern is to be expected—especially when it comes to the C‐cluster as a possible correlate of event files (Takacs, Zink, et al., 2020). The finding that independent spatial activity patterns also constitute dynamics in the S‐cluster, however, also shows that the perceptual or attentional processes involved (as captured by the S‐cluster) are complex. Interestingly, the different ICs constituting the codes of information reflected by the S‐cluster and the C‐cluster show a distinct pattern of the temporal stability of the representational content as revealed by the temporal generalization MVPA results:

For the C‐cluster, both identified ICs revealed clear temporal generalization and stability of the representational content in a time interval between 300 and 650 ms. Such a temporal generalization pattern was, however, also evident when examining Go trials (see supplemental material Figures S5 and S6). The common aspect between Go and Nogo trials in the comparisons performed related to the role of congruent/incongruent stimulus–response mapping processing inducing a more automated or controlled response selection in the respective trials. Thus, it is likely that especially the mode of information processing is reflected the result of the MVPA analysis. For the Nogo trials being the focus of this study, the source localization revealed activity modulations between congruent and incongruent trials in the medial frontal gyrus and the ACC (BA24) and the medial frontal gyrus (BA6) in particular. This is in line with the idea that ACC is activated during tasks involving conflict as induced by incongruent, compared to congruent stimulus–response mappings (Folstein & Van Petten, 2008; Rushworth et al., 2004). However, particularly for the IC2 activity modulations in posterior cingulate cortex were pronounced extending to the regions of the precuneus (BA31 and BA7) in the occipito‐parietal cortex. Moreover, inferior parietal cortices (BA40) were differentially modulated. Activity was stronger in the incongruent than the congruent Nogo trials. The activity pattern associated with the maintenance of the representational content can be explained considering neuroanatomical inter‐relations of the medial frontal cortex and the precuneus. The precuneus (incl. the posterior cingulate cortex) are closely connected regions of the dorsal premotor area including the supplementary motor area (SMA) (Cavada & Goldman‐Rakic, 1989a; Cavada & Goldman‐Rakic, 1989b; Leichnetz, 2001). Interestingly, these areas have previously been implicated in Simon task congruency effects (Herz et al., 2014; Mars et al., 2009; Mückschel et al., 2016; Nachev et al., 2008; Rushworth et al., 2004; Stock et al., 2013) and do also play a key role in response inhibition processes (Bari & Robbins, 2013). Previous work has also shown that these premotor regions are associated with C‐cluster activity modulations in the Simon task (Adelhöfer et al., 2021) and during inhibitory control (Mückschel et al., 2017). Moreover, the C‐cluster has also often been associated with inferior parietal cortex activity modulations (Kleimaker et al., 2020; Opitz et al., 2020; Takacs, Zink, et al., 2020; Wolff et al., 2017), which fits to overarching concepts stating that the inferior parietal cortex plays an essential role updating of mental programmes for response selection (Geng & Vossel, 2013). Importantly, however, a comparison of activity modulations between the ICs revealed that particularly the areas in the precuneus (BA7) and the bilateral inferior parietal cortex (BA40) extending to the temporal cortices (bilaterally) were differentially activated between the isolated Nogo trials C‐cluster ICs. The role of ventral visual stream structures in temporal cortices may be related to the necessity to encode what stimulus is presented (Goodale et al., 2005). According to TEC, event file coding (reflected by the C‐cluster) involves the mapping of stimulus‐related features to the appropriate motor features (Hommel, 2004). Event file dynamics are based on the *identity* of features constituting a stimulus that is bound to a specific response. For example, when the manipulation of facial stimuli modulates event file coding, the ventral stream fusiform face area was found to be involved reflects event file dynamics; when event file coding was modulated by motion stimuli, completely different ventral areas were found to encode event file dynamics (Kühn et al., 2011). Because stimulus information is central to the updating of mental programmes for response selection ventral stream pathways are reasonably involved in event file processing as reflected by the C‐cluster in Nogo trials. The comparison of Nogo trial ICs in the source localization analysis steps shows that even for the ICs being involved in the representation of stimulus–response mapping codes, reflected by the C‐cluster (Ouyang et al., 2015; Ouyang et al., 2017), a hierarchy of spatially independent processes is evident. Structures of the precuneus and the inferior parietal cortex seem to play a more important role than regions in the superior and medial frontal cortex. This is despite all these regions being involved in the processing of inhibitory control processes during Nogo trials and, more precisely, codes specifying rules on how stimulus properties are related to a specific motor response. The reason why this may be the case possibly relates to the findings obtained for the S‐cluster data:

The S‐cluster has been supposed to reflect purely stimulus‐related processes (Ouyang et al., 2015). For the S‐cluster, one IC revealed clear diagonal decoding between 100 and 400 ms after stimulus presentation, but hardly any temporal generalization. The other IC, however, revealed a clear temporal generalization pattern (i.e., off‐diagonal decoding) between 200 and 400 ms after stimulus presentation. For this time interval, the sLORETA analysis revealed that regions of the precuneus (BA31 and BA7) are involved—and thus similar to the C‐cluster. Crucially, however, this was the case earlier in time. Nevertheless, time periods of stable representational content in the S‐cluster (i.e., time interval between 200 and 400 ms) overlap with the C‐cluster (i.e., time interval between 300 and 650 ms). This temporal overlap in the activity of task‐related representations in the same functional neuroanatomical structures (BA7, BA31) makes it likely that stimulus‐related representations are passed to processes that update representations of stimulus–response mapping rules during inhibitory control. The particular relevance of the precuneus and inferior parietal cortex regions (BA7, BA31, BA40) over prefrontal areas (BA24, BA6) for the representation of stimulus–response selection codes in the C‐cluster data may again reflect necessities related to event file coding reflected by the C‐cluster. During event file coding (Opitz et al., 2020; Prochnow et al., 2021; Takacs, Zink, et al., 2020) and stimulus–response mapping, it is particularly important to relate stimulus‐feature information to motor response feature information. These processes are a critical functional of parietal structures (BA7 and BA40) (Geng & Vossel, 2013; Gottlieb, 2007; Gottlieb & Snyder, 2010).

Compared to the Nogo trials discussed above, the ICA of the RIDE clusters in Go trials revealed more ICs. The larger number of extracted ICs in Go trials, compared to Nogo trials might indicate that the cognitive operations for responding depend on a larger spatial scattering of neural processes than for inhibition than for response inhibition.

### 4.1 Implications for cognitive science theory

These insights gained from the combination of different temporal and spatial signal decomposition methods combined with MVPA and source localization have important repercussions on dual‐route models, TEC and how a rather automatic and a controlled route may be implemented and interact on a neural level. As outlined in the introduction, the dual route model (De Jong et al., 1994), supposes that there is an unconditionally automatic process mediated by the “direct route.” The “indirect route” describes the conditional selection of the stimulus features that indicate the appropriate response (Hommel, 2011). In incongruent Simon trials, the automatic and controlled routes are activated and induce a conflict (Chmielewski & Beste, 2017; Hommel, 2011; Keye et al., 2013), which increases controlled processes. This outcome thus reflects an interaction of the direct and indirect routes. To infer what this means in functional terms, Sternberg's additive factor logic is relevant (Scerrati et al., 2017; Sternberg, 1969). Sternberg's Additive Factor Method (AFM) assumes all the processes are in series. According to Sternberg's AFM, if two factors interact, then they are thought to affect the same processing stage. Sets of processes in which some pairs of processes are sequential, and some are concurrent are considered (i.e., the processes are partially ordered). If each process selectively influenced by increments is in the same stage, then the interaction is possible, although not inevitable. In other words, according to Sternberg's AFM, if two factors do not interact, they are said to be additive and are thought to reflect different processing stages—or in neural terms distinct/different (Schweickert et al., 2010). The current study suggests that the latter mostly relates to the representational stability of codes processed in the precuneus and inferior parietal cortex regions (BA7, BA31, BA40) and that particularly stimulus–response mapping operations in these structures are important to consider. However, the fact that there is a complex neural dynamics where different contents of information (i.e., RIDE cluster data) are processed via independent spatial activity pattern (i.e., IC data) suggests that processes that have been conceptualized as dual‐route processes are in fact complex neural dynamics, especially when it comes to the handling of the representational content. This finding is hardly commensurable with a dual‐route account which implies to rather circumscribed processing pathways that are mediating task performance. Considering this, and as mentioned in the introduction, dual‐route models have been criticized (Hommel & Wiers, 2017). Crucially, it has been suggested that the event file concept may be more appropriate to frame processes occurring during Simon Task like conflicts (Hommel, 2011). Importantly, it is also TEC's event file concept which provides a stringent motivation for the concatenation of different EEG signal analysis methods as done in this study (i.e., RIDE, ICA, and MVPA). The event file, also relevant for newer accounts on how perceptual, attentional, and memory processes act in concert to enable goal‐directed acting (Frings et al., 2020), specifies how stimuli are mapped onto a response and also specifies the rules what stimulus features are most relevant to select a motor programme (Hommel, 2004). Crucially, the event file concept assumes distributed processing of perceptual and motor aspects during goal‐directed action control (Hommel, 2004). In line with that, very recent findings reveal that event file processing likely reflects a multi‐region processing of specific fractions of information in the neurophysiological signal (Gholamipourbarogh et al., 2022). Using the event file concept, it has been argued that Simon task effects emerge due to an overlap of stimulus features codes specifying different motor responses (Hommel, 2011). Some of these feature representations are coding task‐relevant information; the other features are coding task‐irrelevant information. When a stimulus is presented, relevant and irrelevant feature codes are activated and lead to the activation of various stimulus–response mapping in an event file. Accordingly, it is unavoidable that “activating a stimulus representation activates overlapping response representations, and vice versa” (Hommel, 2011). Selecting the appropriate response thus requires to reconfigure event file representations when opposing stimulus–response mappings are concomitantly activated in incongruent Simon task trials. The current data show this for Go and Nogo trials. This event file reconfiguration reduces an automated response tendency and thereby increases the likelihood of a successful response inhibition in incongruent Simon Nogo trials. Interestingly, in the last years, several experiments have shown that event files are most likely reflected by C‐cluster activity in the EEG during response selection (Dilcher et al., 2021; Opitz et al., 2020; Takacs, Mückschel, et al., 2020; Takacs, Zink, et al., 2020) and response inhibition (Prochnow et al., 2021). Using EEG‐source localization, all of these studies have revealed that activity differences in inferior parietal cortices are associated with these modulations. All this suggests that the understanding of neurophysiological dynamics subserving representations in inferior parietal cortices is essential to setup neural grounds for overarching cognitive concepts specifying perception‐action integration and the event file concept in particular. It is important to acknowledge that the used methods were able to show that automatic vs. controlled response modes have discriminable representations that can be decoded from neural activity. The convergent pattern in the decoding results for Go and Nogo trials, suggest that it is the congruency information that is coded. However, the results to not provide insights how codes change; that is whether stimulus representations become weaker in the controlled mode during inhibition, or does the rule representation become stronger, and so forth. This will be an important question for future studies, e.g., using representational similarity analysis.

## 5 CONCLUSION

In summary, we show how the representation of the inhibitory control subprocesses are differentially modulated when engaging in response inhibition in a relatively automatic or a controlled mode. On a behavioural level, we replicate previous findings showing that response inhibition was better in incongruent trials than in congruent trials. Regarding neural dynamics, modulations of response inhibition processes by more automated or controlled response selection modes rely on a concomitant coding of stimulus‐related information and rules of how stimulus information has to be related to the appropriate motor programme. Crucially, these fractions of information, which are encoded at the same time in the neurophysiological signal, are based on two independent spatial neurophysiological activity patterns, also showing differences in the temporal stability of the representational content. Source localization analysis revealed that the precuneus and inferior parietal cortex regions (BA7, BA31, BA40) are more relevant than prefrontal areas (BA24, BA6) for the representation of stimulus–response selection codes. This may be a consequence of the timing of stimulus‐related and stimulus–response‐related representational contents. The work shows how a concatenation of different EEG signal analysis methods, capturing distinct aspects of neural dynamics, can be connected to the cognitive science theory of action control.

## AUTHOR CONTRIBUTIONS

All authors had full access to the data, gave final approval for publication and agree to be held accountable for the work performed therein. *Conceptualization*, Negin Gholamipourbarogh, Christian Beste, Moritz Mückschel; *Software*, Negin Gholamipourbarogh, Moritz Mückschel, *Investigation*, Filippo Ghin, Ann‐Kathrin Stock; *Formal Analysis*, Negin Gholamipourbarogh; *Writing – Original Draft*, Negin Gholamipourbarogh, Christian Beste, Moritz Mückschel; *Writing – Reviewing and Editing*, all authors; *Visualization*, Negin Gholamipourbarogh; *Supervision*, Christian Beste; *Funding Acquisition*, Christian Beste, Ann‐Kathrin Stock.

## FUNDING INFORMATION

This work was partly supported by Grants from the Deutsche Forschungsgemeinschaft (DFG) SFB TRR 265, FOR 2790, and FOR 2698.

## CONFLICT OF INTEREST

The authors declare no conflicts of interest.

## Supporting information


**FIGURE S1** The scalp topography plots of the original ICs for the RIDE C‐cluster congruent condition in the Nogo data group.
**FIGURE S2** The scalp topography plots of the original ICs for the RIDE C‐cluster incongruent condition in the Nogo data group.
**FIGURE S3** The scalp topography plots of the original ICs for the RIDE S‐cluster congruent condition in the Nogo data group.
**FIGURE S4** The scalp topography plots of the original ICs for the RIDE S‐cluster incongruent condition in the Nogo data group.
**FIGURE S5** MVPA results for similar IC pair of the RIDE S‐cluster for Go condition trials. Left figures show IC scalp topographies from the CORRMAP processing results for congruent (top) and incongruent (bottom) conditions. The scalp topographies reveal the weighting matrices of each IC. Middle curves reveal the binary classification performance. For the binary classification, the shaded error bars represent standard deviation. Right figures show the temporal generalization of similar independent components. All time points are in milliseconds.
**FIGURE S6** MVPA results for three similar IC pairs of the RIDE C‐cluster for Go condition trials. Left figures IC scalp topographies from the CORRMAP processing results for congruent (top) and incongruent (bottom) conditions. The scalp topographies reveal the weighting matrices of each IC. Middle curves reveal the binary classification performance. For the binary classification, the shaded error bars represent standard deviation. Right figures show the temporal generalization of similar independent components. All time points are in milliseconds. It should be noted that CORRMAP extracted five similar IC pairs for the C cluster, but for the first two component pairs, MVPA results were not significant.

## Data Availability

The data that support the findings of this study are available from the corresponding author upon reasonable request.
