# Supplementary material for: Evidence for independent representational contents in inhibitory control subprocesses associated with frontoparietal cortices
Source: Hum Brain Mapp. 2022 Oct 31;44(3):1046–61. doi: 10.1002/hbm.26135 (PMC9875938; doi:10.1002/hbm.26135)
Supplement: Supplementary file 1 — FIGURE S1 The scalp topography plots of the original ICs for the RIDE C‐cluster congruent condition in the Nogo data group. FIGURE S2 The scalp topography plots of the original ICs for the RIDE C‐cluster incongruent condition in the Nogo data group. FIGURE S3 The scalp topography plots of the original ICs for the RIDE S‐cluster congruent condition in the Nogo data group. FIGURE S4 The scalp topography plots of the original ICs for the RIDE S‐cluster incongruent condition in the Nogo data group. FIGURE S5 MVPA results for similar IC pair of the RIDE S‐cluster for Go condition trials. Left figures show IC scalp topographies from the CORRMAP processing results for congruent (top) and incongruent (bottom) conditions. The scalp topographies reveal the weighting matrices of each IC. Middle curves reveal the binary classification performance. For the binary classification, the shaded error bars represent standard deviation. Right figures show the temporal generalization of similar independent components. All time points are in milliseconds. FIGURE S6 MVPA results for three similar IC pairs of the RIDE C‐cluster for Go condition trials. Left figures IC scalp topographies from the CORRMAP processing results for congruent (top) and incongruent (bottom) conditions. The scalp topographies reveal the weighting matrices of each IC. Middle curves reveal the binary classification performance. For the binary classification, the shaded error bars represent standard deviation. Right figures show the temporal generalization of similar independent components. All time points are in milliseconds. It should be noted that CORRMAP extracted five similar IC pairs for the C cluster, but for the first two component pairs, MVPA results were not significant. [file HBM-44-1046-s001.docx]

**Supplementary Material**

**Evidence for independent representational contents in inhibitory control subprocesses associated with fronto-parietal cortices**

Negin Gholamipourbarogh, Filippo Ghin, Moritz Mückschel, Christian Frings, Ann-Kathrin Stock, Christian Beste


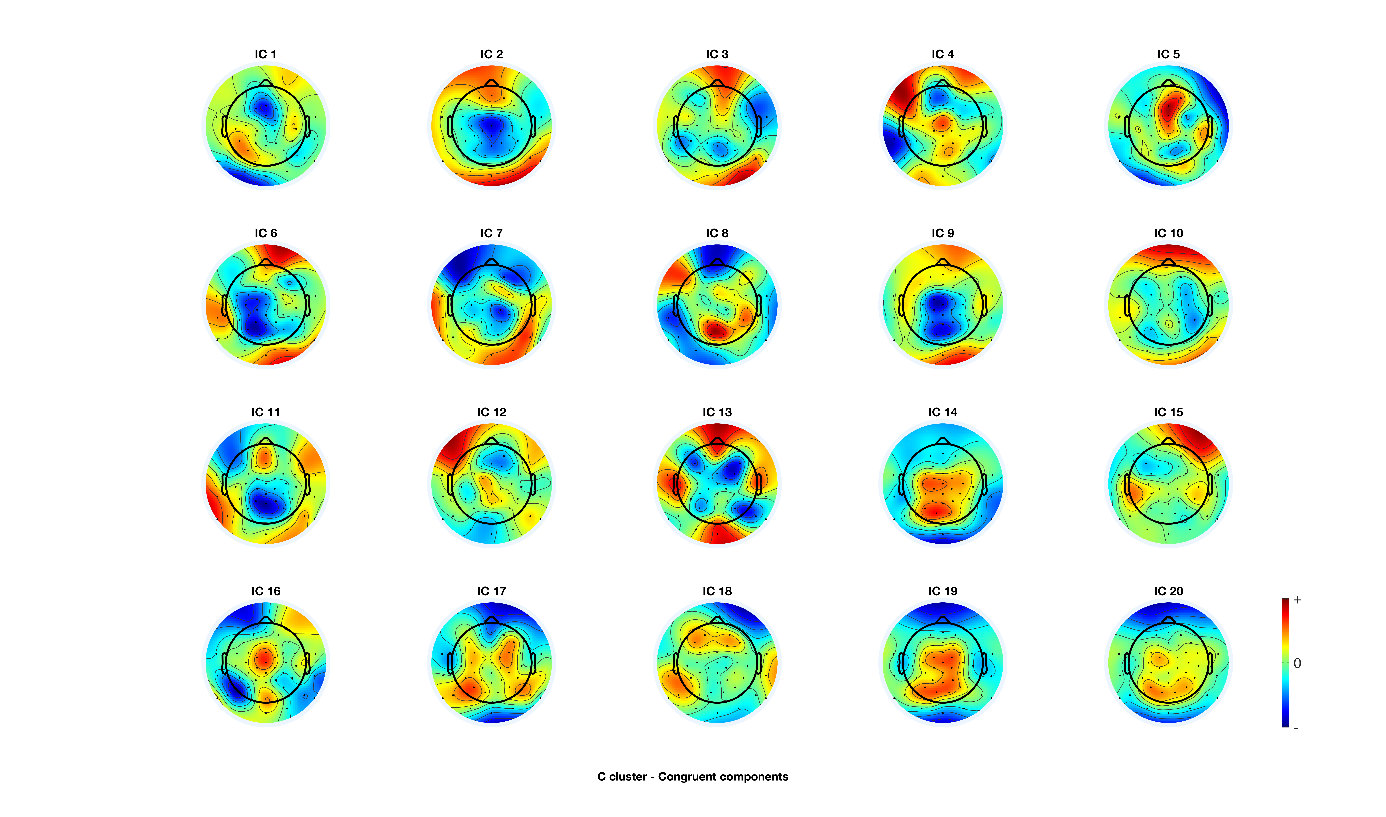


Figure S1- The scalp topography plots of the original ICs for the RIDE C-cluster congruent condition in the Nogo data group.


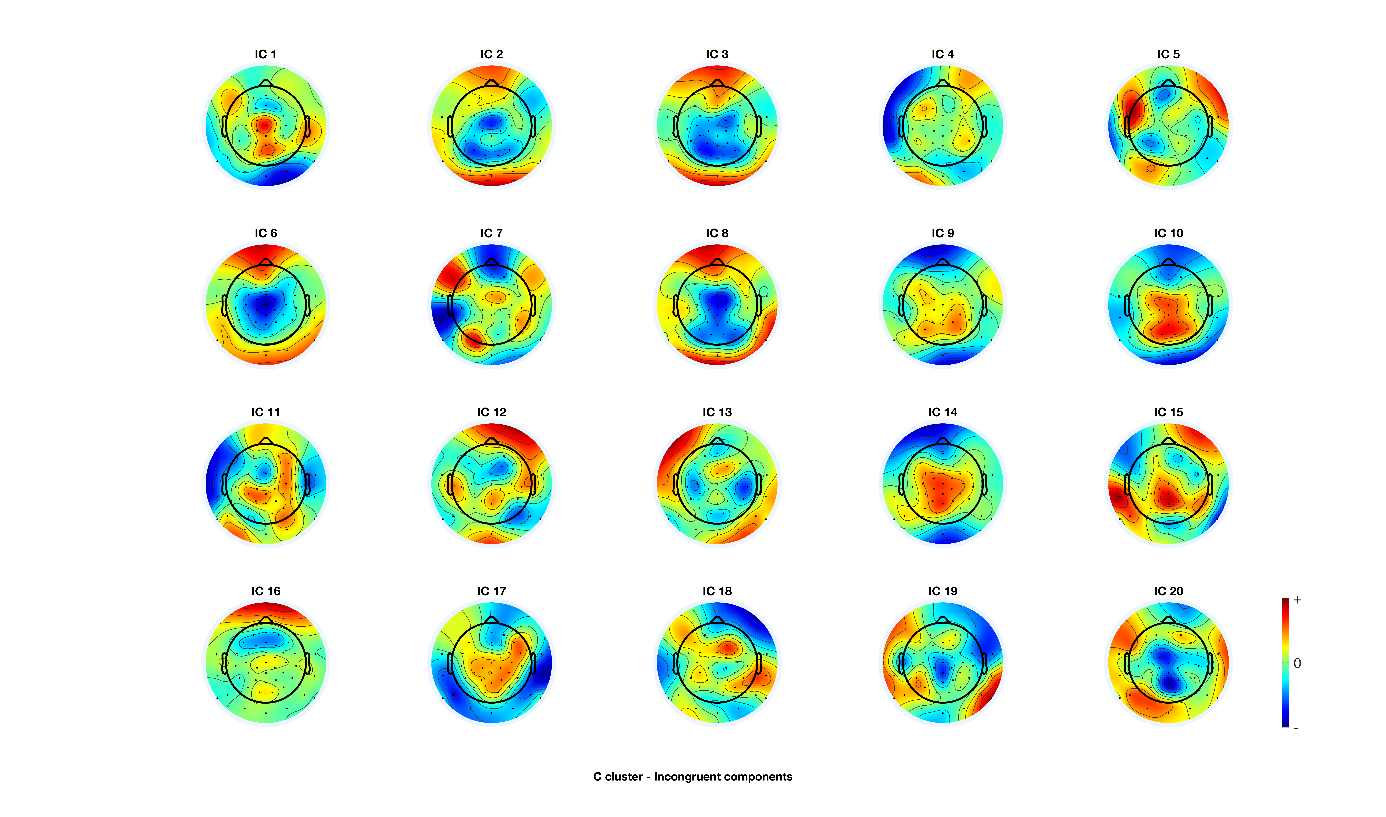


Figure S2- The scalp topography plots of the original ICs for the RIDE C-cluster incongruent condition in the Nogo data group.


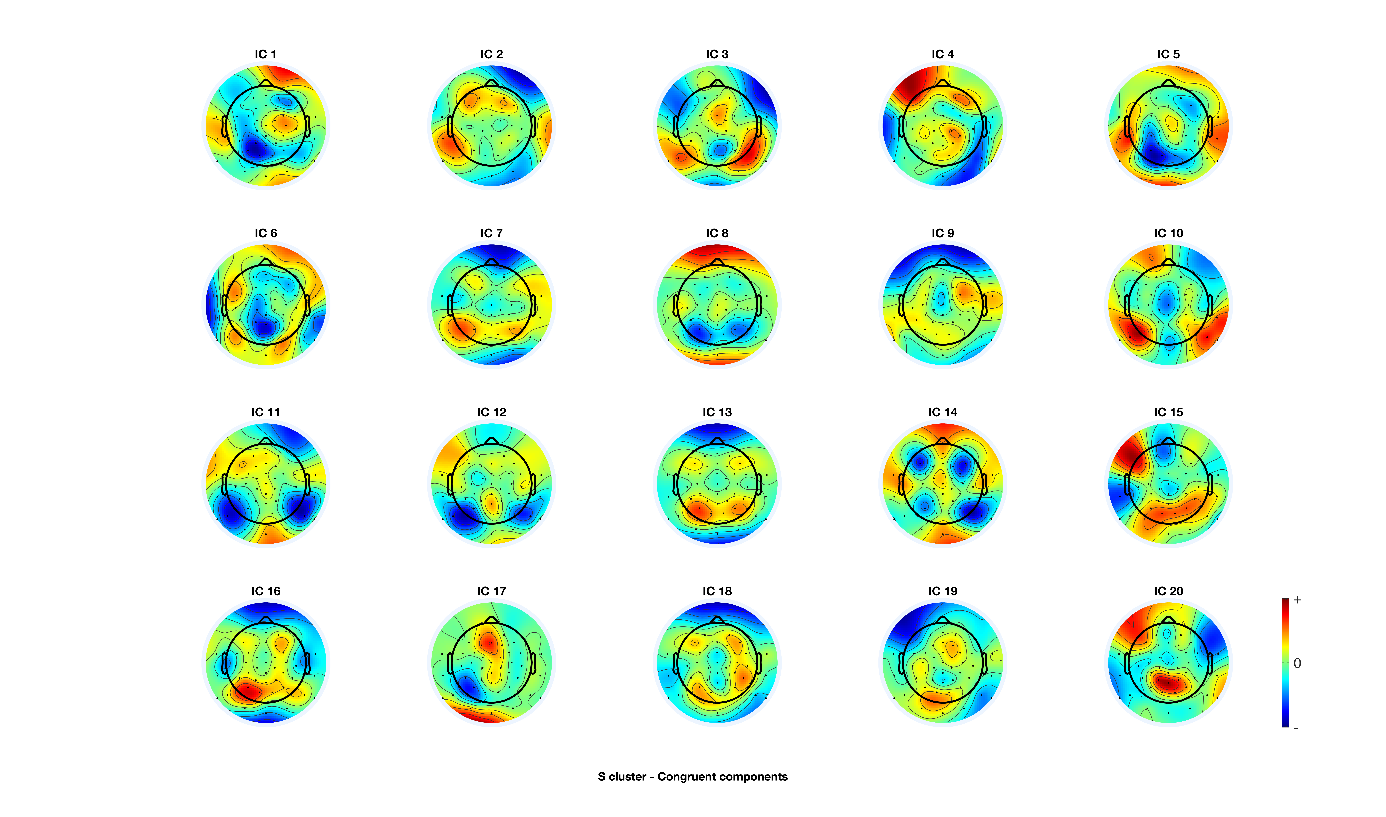


Figure S3- The scalp topography plots of the original ICs for the RIDE S-cluster congruent condition in the Nogo data group.


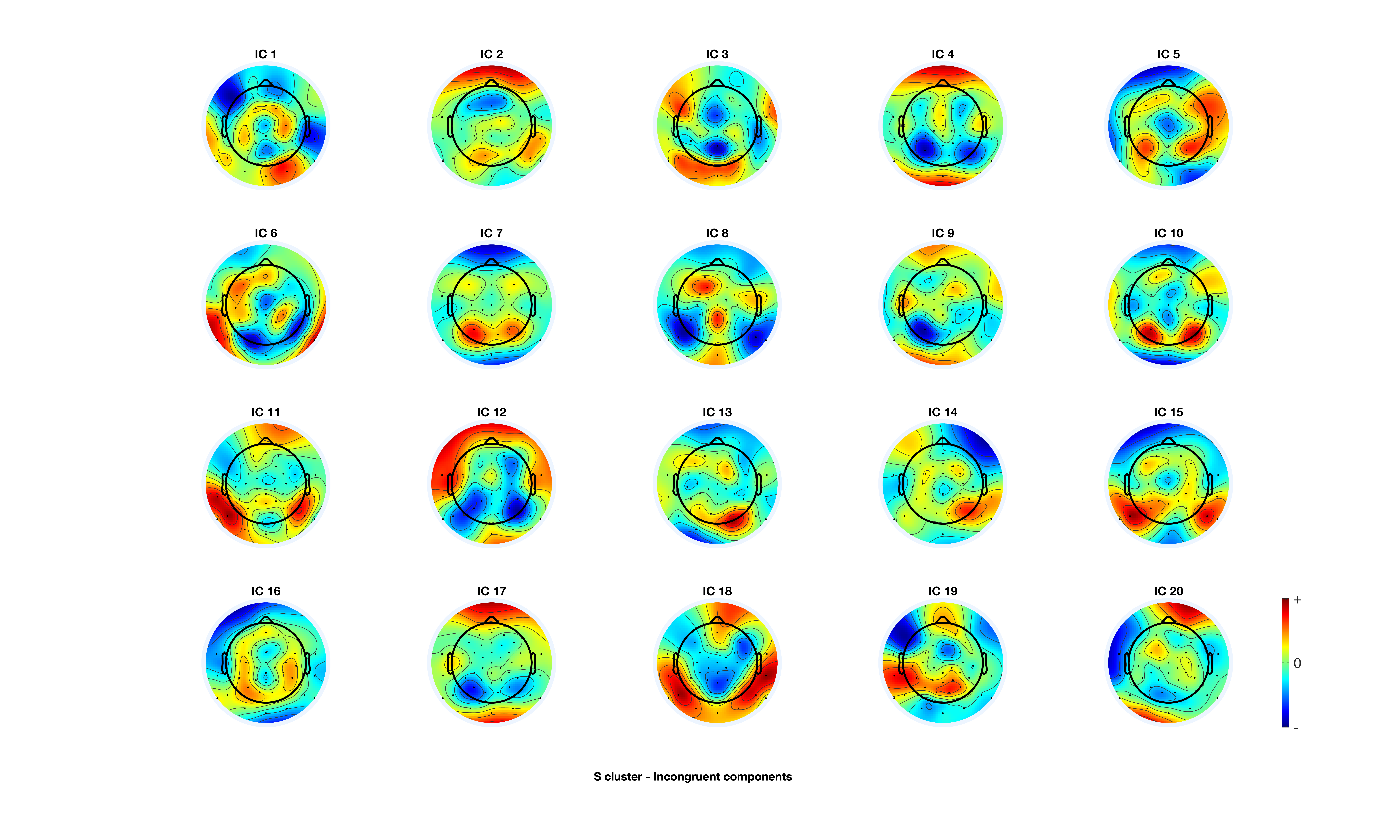


Figure S4- The scalp topography plots of the original ICs for the RIDE S-cluster incongruent condition in the Nogo data group.

**Go condition results**


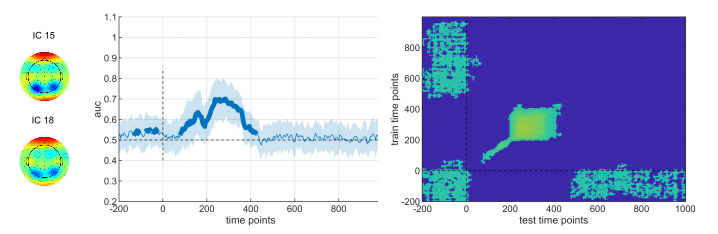


Figure S5- MVPA results for similar IC pair of the RIDE S-cluster for Go condition trials. Left figures show IC scalp topographies from the CORRMAP processing results for congruent (top) and incongruent (bottom) conditions. The scalp topographies reveal the weighting matrices of each IC. Middle curves reveal the binary classification performance. For the binary classification, the shaded error bars represent standard deviation. Right figures show the temporal generalization of similar independent components. All time points are in milliseconds.

**
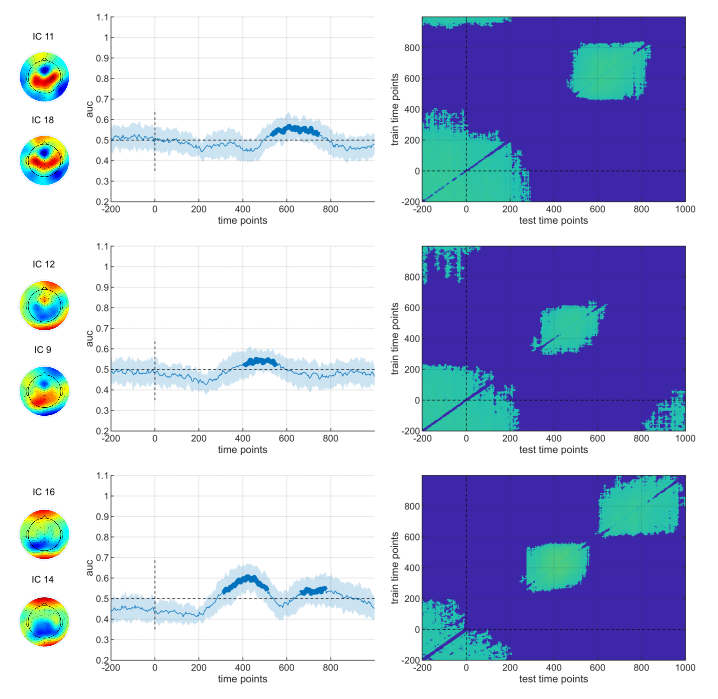
**

Figure S6- MVPA results for three similar IC pairs of the RIDE C-cluster for Go condition trials. Left figures IC scalp topographies from the CORRMAP processing results for congruent (top) and incongruent (bottom) conditions. The scalp topographies reveal the weighting matrices of each IC. Middle curves reveal the binary classification performance. For the binary classification, the shaded error bars represent standard deviation. Right figures show the temporal generalization of similar independent components. All time points are in milliseconds. It should be noted that CORRMAP extracted five similar IC pairs for the C cluster, but for the first two component pairs, MVPA results were not significant.
